# Supplementary material for: Engineering and Implementation of Synthetic Molecular Tools in the Basidiomycete Fungus Ustilago maydis
Source: J Fungi (Basel). 2023 Apr 17;9(4):480. doi: 10.3390/jof9040480 (PMC10140897; doi:10.3390/jof9040480)
Supplement: Supplementary file 1 [file jof-09-00480-s001.zip › jof-2296445-supplementary.pdf]

Supplementary Information

Engineering and Implementation of Synthetic Molecular Tools in the Basidiomycete Fungus *Ustilago maydis*

Nicole Heucken [1,†], Kun Tang [1,†], Lisa Hüsemann [1,†], Natascha Heßler [2], Kira Müntjes [2], Michael Feldbrügge [2], Vera Göhre [2,3,*] and Matias D. Zurbriggen [1,3,*]

[1] Institute of Synthetic Biology, University of Düsseldorf, 40225 Düsseldorf, Germany
[2] Institute of Microbiology, University of Düsseldorf, 40225 Düsseldorf, Germany
[3] CEPLAS—Cluster of Excellence on Plant Sciences, 40225 Düsseldorf, Germany
* Correspondence: vera.goehre@hhu.de (V.G.); matias.zurbriggen@uni-duesseldorf.de (M.D.Z.); Tel.: +49-211-81-11529 (V.G.); +49-211-81-15015 (M.D.Z.)
† These authors contributed equally to this work.

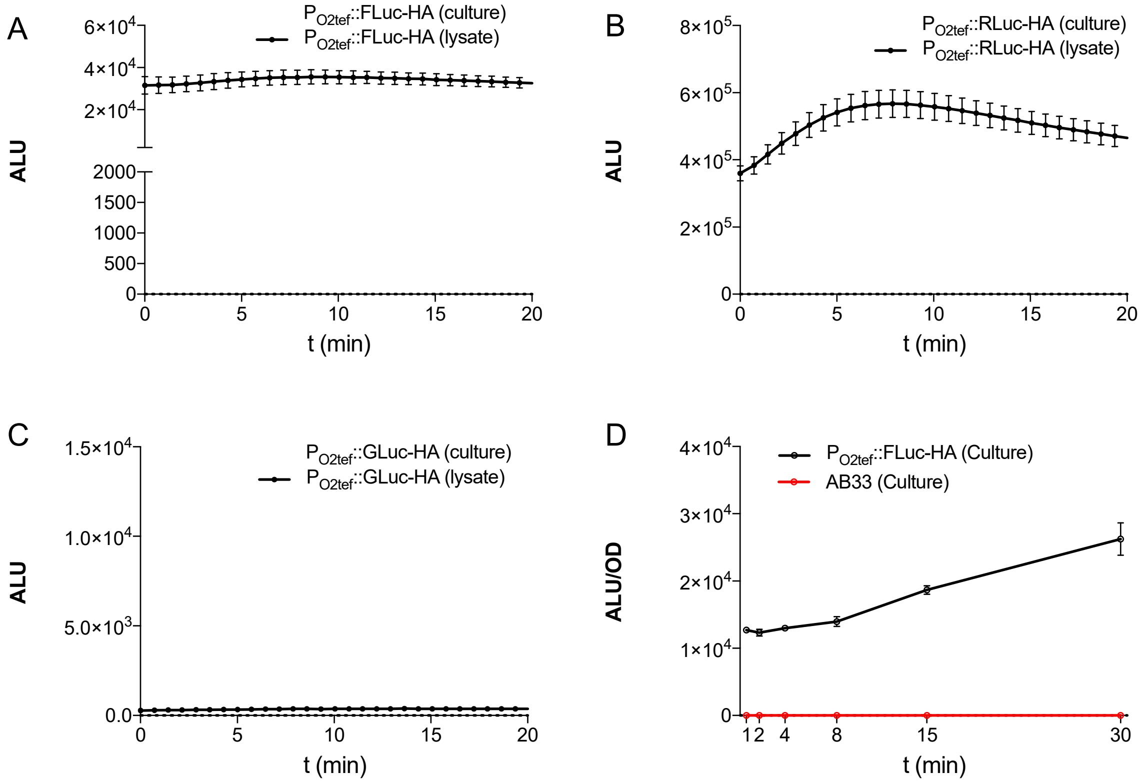

Figure S1. Time course of luciferase activities in lysates and whole cell cultures. Lysates of 2 mL cultures and the respective whole cell cultures, OD_{600} = 0.5, of constitutively expressing luciferase strains were analyzed for their luminescence over 20 minutes after addition of substrates. FLuc (A), RLuc (B), and GLuc (C) luminescence is given in absolute luminescence units. (D) For the reporter measurement, 80 µL of the whole cell culture were transferred to 96-well assay plates and the firefly substrate was added directly and measured in 1, 2, 4, 8, 15- and 30-min. Values are normalized to an OD_{600} of 0.5. Error bars represent the SEM for this individual experiment with n=3.

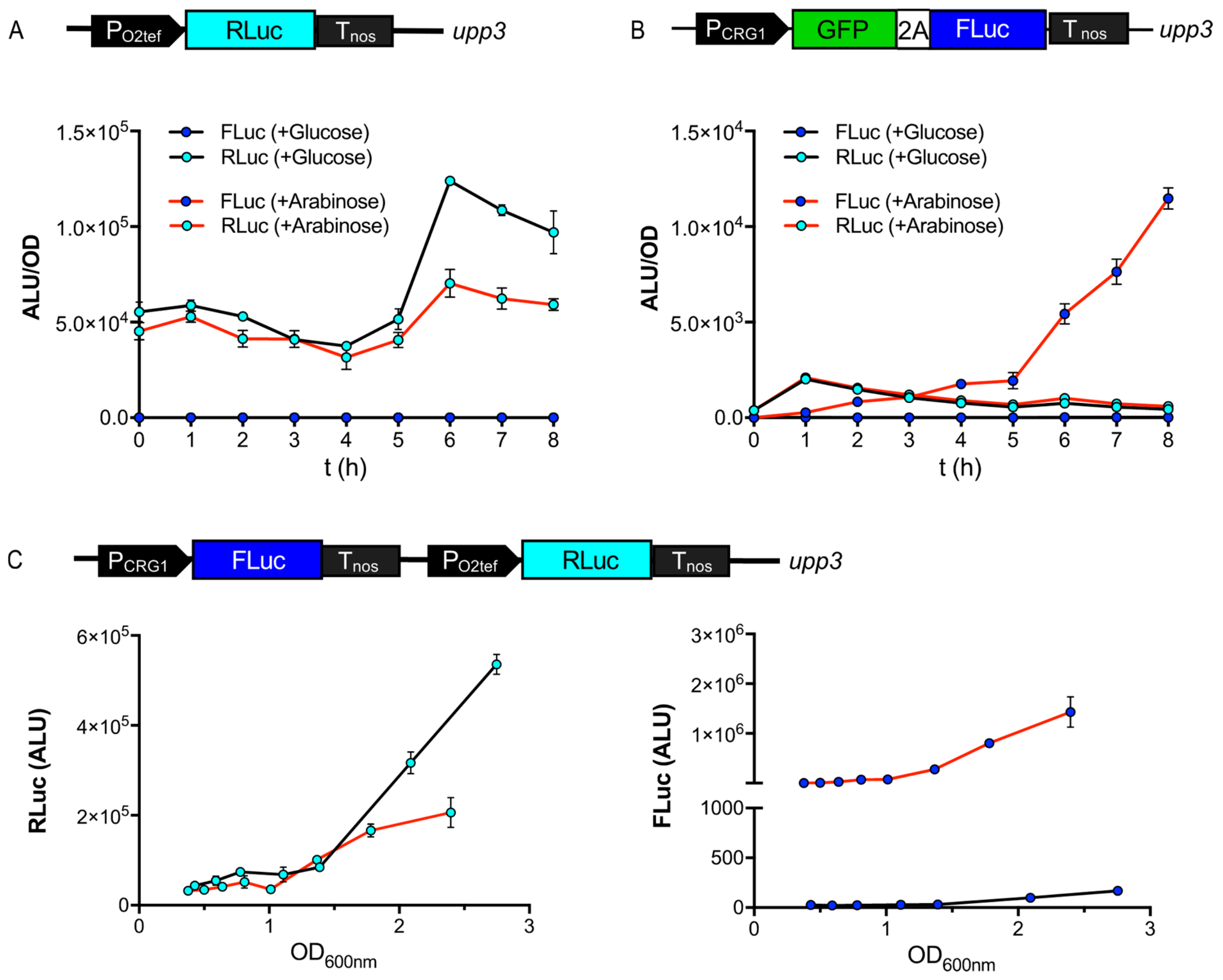

Figure S2. Normalization of gene expression in *U. maydis*. (A,B) single luminescence reporter strains. RLuc under control of the constitutive promotor P_{O2tef} (A, sLHNH005) and FLuc (as part of a bicistronic construct) under control of the arabinose-inducible promotor P_{crg1} (B, UMa3212) were cultured in CM-glucose or arabinose medium for absolute luminescence measurements. Cultures were grown overnight in CM-Glucose, at timepoint 0 determination started (black line), or cultures were shifted to CM-Arabinose (red line). 2 mL cultures were taken and lysed every hour, and analyzed for FLuc (blue dots) and RLuc (cyan dots) luminescence (normalized to an OD_{600} = 0.5). (C) Dual-luminescence reporter strain (sNH039, detail information in Fig.2 and Table S3) were cultured in CM-glucose (black line) or arabinose (red line) medium. RLuc values were correlated with the OD_{600} during exponential growth in both conditions, while FLuc activity only increased upon the arabinose induction.

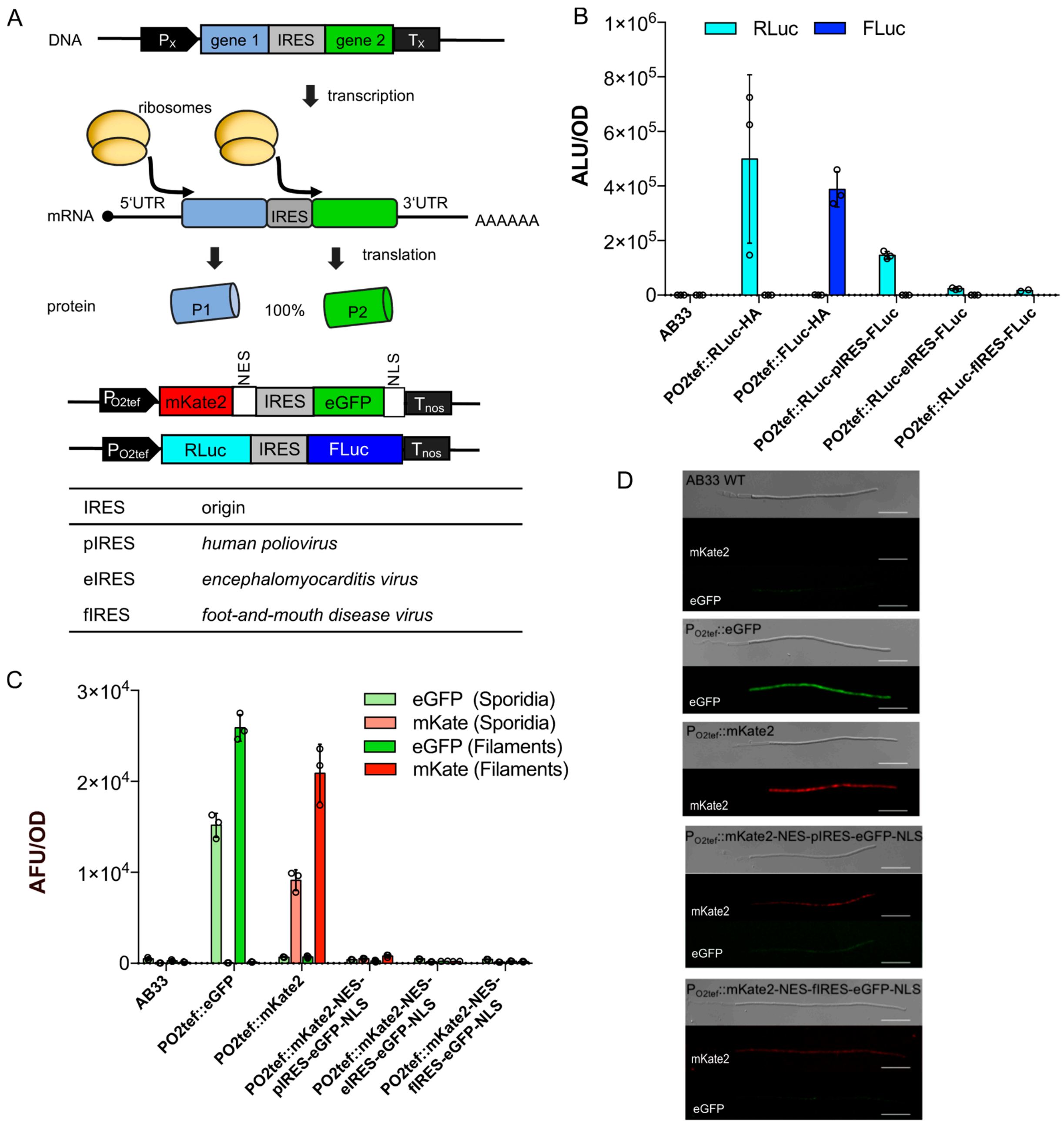

Figure S3. Establishment of IRES sequences in *U. maydis*. (A) Schematic representation of the IRES based bi-reporters: Genes 1 and 2 were expressed under the control of the constitutive promotor Px and are separated by IRES sequence. IRES bi-reporters strains transformed into the *upp3* locus of AB33. (B) Luminescence of whole cell lysates from 2 mL cultures, OD_{600} = 0.5, of the indicated strains is shown (absolute luminescence units). (C) Fluorescence intensity of control and IRES strains measured in cultures of an OD_{600} = 0.5 in sporidia, and six hours after induction of filamentous growth in a plate reader in absolute fluorescence units (AFU). In B and C, data are presented as mean values +/- SEM, n=3 individual experiments. (D) Microscopic analysis of WT AB33, mKate2, and eGFP positive controls and pIRES and fIRES strains six hours after induction of filamentous growth. Scale-bars represent 20 µm.

Table S1. Generation and description of plasmids used in this work.
All plasmids are constructed with AQUA or Gibson assembly cloning (Gibson et al., 2009; Beyer et al., 2015) if not indicated otherwise. Grey and bold: plasmids used in strain generation; white: intermediate cloning plasmids.

Plasmid			Description	Reference
**pLHNH031**			**P_{O2tef}-GLuc-HA-nosT**<br>Vector encoding GLuc-HA under the control of P_{O2tef}. GLuc was amplified from pLHNH018 with oNH016 and oNH117 adding an HA-tag c-terminally to GLuc. pLHNH001 was digested with MfeI and BglII, and assembled via AUQA cloning.	this work<br>(for generating strain sLHNH006)
↳	pLHNH018		Vector encoding the synthesized codon optimized GLuc	this work
↳	pLHNH001		P_{O2tef}-GLuc-NLS-nosT<br>Vector encoding GLuc-NLS under the control of P_{O2tef}. pUMa3132 and pLHNH029 were digested with SfbI and AflII, and ligated with QuickLigase.	this work
	↳	pLHNH029	Vector encoding the synthesized P_{O2tef} and codon optimized GLuc-NLS	this work
	↳	pUMa3132	P_{O2tef}-eGFP-nosT-NatR<br>Vector encoding eGFP under the control of P_{O2tef} and the Nourseothricin resistance cassette for integration into the *upp3*-locus	Lee et al., 2020 (ref [10] in main text)
**pLHNH030**			**P_{O2tef}-RLuc-HA-nosT**<br>Vector encoding RLuc-HA under the control of P_{O2tef}. pLHNH001 was digested with MfeI and BglII, RLuc was amplified from pLHNH019 with oNH020 and oNH116 adding an HA-tag c-terminally to RLuc.	this work<br>(for generating strain sLHNH005)
↳	pLHNH019		Vector encoding the synthesized codon optimized RLuc	this work
**pLHNH032**			**P_{O2tef}-SEAP-HA-nosT**<br>Vector encoding SEAP-HA under the control of P_{O2tef}. pLHNH001 was digested with MfeI and BglII, SEAP was amplified from pLHNH034 with oNH048 and oNH132, adding an HA-tag c-Terminally to SEAP.	this work<br>(for generating strain sLHNH007)
↳	pLHNH034		P_{O2tef}-SEAP-nosT<br>Vector encoding SEAP under the control of P_{O2tef}. pLHNH001 was digested with MfeI and BglII, SEAP n-term was amplified from pLHNH020 with oLH017 and oLH021, SEAP c-term was amplified from pLHNH035 with oNH130 and oNH131. SEAP parts were fused via PCR with oNH048 and oNH131.	this work
	↳	pLHNH020	Vector encoding the synthesized codon optimized SEAP-nTerm	this work
	↳	pLHNH035	Vector encoding the synthesized codon optimized SEAP-cTerm	this work
**pLHNH033**			**P_{O2tef}-FLuc-HA-nosT**<br>Vector encoding FLuc-HA under the control of P_{O2tef}. pLHNH001 was digested with MfeI and BglII, FLuc was amplified from pLHNH017 with oNH012 and oNH119 adding an HA-Tag c-terminally to FLuc.	this work<br>(for generating strain sLHNH008)
↳	pLHNH017		Vector encoding the synthesized codon optimized FLuc	this work
**pNH054**			**P_{CGR}-FLuc-nosT-P_{O2tef}-RLuc-nosT**<br>Bicistronic vector encoding FLuc under the control of the inducible P_{CRG} and RLuc under the control of P_{O2tef}.	this work<br>(for generating strain sNH039)

	pLHNH030 was digested with SbfI. FLuc was amplified from pLHNH017 using oLH014 and oLH018, nosT was amplified from pUMa3132 using oNH715 and oNH144. FLuc and nosT were fused via PCR using oNH717 and oNH144. P_{CRG} was amplified from pUMa4175 using oNH718 and oNH719. FLuc-nosT and P_{CRG} were fused via PCR using oNH720 and oNH716.	
↳ pUMa4175	P_{CRG}-5’UTR-rrm4-eGFP-e’UTR-nosT<br>Plasmid encoding a fusion of rrm4 and eGFP under the control of the inducible CRG promoter.	this work
**pNH009**	**P_{O2tef}-mKate2-NES-pIRES-eGFP-NLS-nosT**<br>Bicistronic vector encoding mKate2-NES and eGFP-NLS under the control of P_{O2tef}. pLHNH001 was digested with MfeI and PacI, mKate2 was amplified from pLHNH015 with oNH058 and oNH122, eGFP was amplified from pLHNH015 with oNH205 and oNH057, human polio virus IRES was amplified from pKM006 with oNH124 and oNH125, mKate2, pIRES and eGFP were fused via PCR using oNH008 and oNH123.	this work<br>(for generating strain sLHNH009)
↳ pKM006	tetO13-422 bp-PhCMVmin-SEAP-pvIRES-pA<br>Vector encoding SEAP-pvIRES under the control of a modified Tet between inducible promoter	Müller et al., 2013 (ref [20] in main text)
**pNH010**	**P_{O2tef}-mKate2-NES-eIRES-eGFP-NLS-nosT**<br>Bicistronic vector encoding mKate2-NES and eGFP-NLS under the control of P_{O2tef}. pLHNH001 was digested with MfeI and PacI, mKate2 was amplified from pLHNH015 with oNH058 and oNH122, eGFP was amplified from pLHNH015 with oNH205 and oNH057, Encephalomyocarditis virus IRES was amplified from pLHNH036 with oNH126 and oNH127, mKate2, eIRES and eGFP were fused via PCR using oNH008 and oNH123.	this work<br>(for generating strain sLHNH010)
↳ pLHNH036	Encephalomyocarditis virus IRES	this work
**pNH011**	**P_{O2tef}-mKate2-NES-fIRES-eGFP-NLS-nosT**<br>Bicistronic vector encoding mKate2-NES and eGFP-NLS under the control of P_{O2tef}. pLHNH001 was digested with MfeI and PacI, mKate2 was amplified from pLHNH015 with oNH058 and oNH122, eGFP was amplified from pLHNH015 with oNH205 and oNH057, Foot-and-mouthdisease virus IRES was amplified from pLHNH037 with oNH128 and oNH129, mKate2, fIRES and eGFP were fused via PCR using oNH008 and oNH123.	this work<br>(for generating strain sLHNH011)
↳ pLHNH037	Foot-and-mouthdisease virus IRES	this work
**pNH026**	**P_{O2tef}- RLuc-pIRES-FLuc-nosT**<br>Bicistronic vector encoding RLuc and FLuc under the control of P_{O2tef}. pLHNH001 was digested with MfeI and AscI, RLuc was amplified from pLHNH019 using oligos oLH015 and oNH181, pIRES was amplified from pKM006 using oligos oNH612 and oNH613, RLuc and pIRES were fused via PCR using oligos oNH020 and oNH613. FLuc was amplified from pLHNH017 using oligos oNH175 and oNH174. FLuc and the RLuc-pIRES fusion were fused via PCR using oligos oNH623 and oNH624.	this work<br>(for generating strain sNH001)
**pNH028**	**P_{O2tef}-RLuc-eIRES-FLuc-nosT**	this work

	Bicistronic vector encoding RLuc and FLuc under the control of P_{O2tef}. pLHNH001 was digested with MfeI and AscI, RLuc was amplified from pLHNH019 using oligos oLH015 and oNH178, eIRES was amplified from pLHNH036 using oligos oNH614 and oNH615, RLuc and eIRES were fused via PCR using oligos oNH020 and oNH615. FLuc was amplified from pLHNH017 using oligos oNH177 and oNH174. FLuc and the RLuc-IeRES fusion were fused via PCR using oligos oNH623 and oNH624.	(for generating strain sNH003)
**pNH029**	**P_{O2tef}-RLuc-fIRES-FLuc-nosT**<br>Bicistronic vector encoding RLuc and FLuc under the control of P_{O2tef}. pLHNH001 was digested with MfeI and AscI, RLuc was amplified from pLHNH019 using oligos oLH015 and oNH180, fIRES was amplified from pLHNH037 using oligos oNH616 and oNH617, RLuc and fIRES were fused via PCR using oligos oNH020 and oNH617. FLuc was amplified from pLHNH017 using oligos oNH179 and oNH174. FLuc and RLuc-fIRES fusion were fused via PCR using oligos oNH623 and oNH624.	this work<br>(for generating strain sNH004)
**pNH012**	**nosT-NES-mKate2-$P_{hCMVmin}$-CMVenhancer(5'->3')-$P_{hCMVmin}$-eGFP-NLS-nosT**<br>Bicistronic vector encoding mKate2-NES and eGFP-NLS under the control of a bidirectional P_{CMV}. pNH012a was digested with MfeI; mKate2 was amplified from pUMa2977 using oligos oNH139 and oNH141.	this work<br>(for generating strain sNH005)
↳ pNH012a	pNH030 (see below) was digested with AscI, eGFP was amplified from pUMa3132 using oligos oNH136 and oNH123. Parts were assembled via AQUA cloning.	this work
pUMa2977	Vector containing a synthesized dicodon-optimized red fluorescent protein mKate2 with an HA-tag.	Mu¨ntjes et al., 2020 (ref [15] in main text)
**pNH013**	**nosT-NES-mKate2-$P_{hCMVmin}$-CMVenhancer(5'<-3')-$P_{hCMVmin}$-eGFP-NLS-nosT**<br>Bicistronic vector encoding mKate2-NES and eGFP-NLS under the control of a bidirectional P_{CMV}. pNH013a was digested with MfeI, eGFP was amplified from pUMa3132 using oligos oNH659 and oNH660.	this work<br>(for generating strain sNH006)
↳ pNH013a	pNH012 was digested with AscI, mKate2 was amplified from pUMa2977 using oligos oNH657 and oNH658. Parts were assembled via Aqua cloning.	this work
**pNH014**	**nosT-NES-mKate2-$P_{mfa1min}$-(prf1)$_4$(5'->3')-$P_{mfa1min}$-GFP-NLS-nosT**<br>Bicistronic vector encoding mKate2-NES and eGFP-NLS under the control of a bidirectional P(prf1)$_4$-mfa1min. pNH014a was digested with AscI, eGFP was amplified from pUMa3132 with oNH721 and oNH123.	this work<br>(for generating strain sNH007)
↳ pNH014a	pNH032 was digested with MfeI; mKate2 was amplified from pUMa2977 with oNH141 and oNH153, parts were assembled via AQUA cloning.	this work
**pNH015**	**nosT-NES-mKate2-$P_{mfa1min}$-(prf1)4(5'<-3')-$P_{mfa1min}$-GFP-NLS-nosT**<br>Bicistronic vector encoding mKate2-NES and eGFP-NLS under the control of a bidirectional P(prf1)4-mfa1min. pNH015a was digested with AscI, mKate2 was amplified from pUMa2977 with oNH722 and oNH658.	this work<br>(for generating strain sNH008)

↳	pNH015a		pNH032 was digested with MfeI, eGFP was amplified from pUMa3132 with oNH152 and oNH660. Parts were assembled via AQUA cloning.	this work
**pNH030**			**nosT-RLuc-$P_{hCMVmin}$-CMVenhancer(5'->3')-$P_{hCMVmin}$-FLuc-nosT**<br>Bicistronic vector encoding RLuc and FLuc under the control of a bidirectional P_{CMV}. pNH030a was digested with SbfI and MfeI, RLuc was amplified from pLHNH019 using oligos oLH019 and oNH196, a $P_{hCMVmin}$ was added via PCR using oligos oNH197 and oNH140, nosT was amplified from pUMa3132 using oligos oNH158 and oNH144, $P_{hCMVmin}$-RLuc and nosT were fused via PCR using oligos oNH169 and oNH143.	this work<br>(for generating strain sNH011)
↳	pNH030a		pLHNH001 was digested with MfeI and AscI; FLuc was amplified from pLHNH017 using oligos oNH198 and oLH018, P_{CMV} was amplified from pHB109 using oligos oNH137 and oNH135, P_{CMV} and FLuc were fused via PCR using oligos oNH174 and oNH625. Parts were assembled via AQUA cloning.	this work
	↳	pHB109	P_{CMV}-IE-PhyB-mCherry-NES-pA<br>Vector encoding PhyB-mCherry-NES under the control of the hCMV immediate early promoter.	Beyer et al., 2015 (ref [22] in main text)
**pNH031**			**nosT-RLuc-$P_{hCMVmin}$-CMVenhancer(5'<-3')-$P_{hCMVmin}$-FLuc-nosT**<br>Bicistronic vector encoding RLuc and FLuc under the control of a bidirectional P_{CMV}. pNH031a was digested with MfeI. FLuc was amplified from pLHNH017 using oligos oNH184 and oNH647.	this work<br>(for generating strain sNH012)
↳	pNH031a		pNH030 was digested with AscI. RLuc was amplified from pLHNH019 using oligos oNH646 and oNH645. Parts were assembled via Aqua cloning.	this work
**pNH032**			**nosT-RLuc-$P_{mfa1min}$-$(prf1)_4$(5'->3')-$P_{mfa1min}$-Fluc-nosT**<br>Bicistronic vector encoding Rluc and Fluc under the control of a bidirectional P(prf1)4-mfa1min. pNH032b was digested with MfeI and SbfI. nosT was amplified from pUMa3132 with oNH144 and oNH142, Rluc was amplified from pLHNH019 with oNH627 and oNH197. nosT and Rluc were fused via PCR using oNH144 and oNH628. The resulting fragment was again amplified using oNH143 and oNH157 to add overhangs to the backbone.	this work<br>(for generating strain sNH013)
↳	**pNH032b**		pNH032a was digested with MfeI and PacI, P_{OMA} was amplified from pUMa2675 with oNH626 and oNH696 and parts were assembled via AQUA cloning. 4 repeats of the prf1 enhancer were lost during cloning.	this work
	↳	pNH032a	pLHNH001 was digested with PacI and AscI, Fluc was amplified from pLHNH017 with oNH695 and oNH174. Parts were assembled via AQUA cloning.	this work
	↳	pUMa2675	P_{OMA}-eGFP-nosT<br>Vector encoding eGFP under the control of a prf1 operator-mfa1min promoter (P_{OMA} = (prf1)8-$P_{mfa1min}$).	this work
**pUMa2986**			**pep4D: Potef-mKate2-Tnos-natR**<br>Vector encoding mKate2 under the control of P_{O2tef} and the Nourseothricin resistance cassette for integration into the *pep4*-locus. pUMa2977 was digested with AscI and NotI to integrate mKate2 into a backbone for expression in the *pep4* locus.	this work<br>(for generating strain UMa1987)

Table S2. oligonucleotides used in this work.

Oligo	Sequence (5′--3′)	Description
oLH014	ATGGAGGACGCCAAGAA	Fw FLuc
oLH015	ATGACCAGCAAGGTCTAC	Fw RLuc
oLH017	ATGGTGCTCGGTCCTT	Fw SEAP
oLH018	TTAGACGGCGATCTTGC	Rev FLuc
oLH019	TTACTGCTCGTTCTTGAGC	Rev RLuc
oLH021	TTAGTCGATGTCCATGTTCG	Rev SEAP
oNH008	CGGGATCCCCCGGGCTGCAGGAATTCGATCCCCAATTGATGGTGTCGGAGCTCAT	Fw mKate2
oNH012	CGGGATCCCCCGGGCTGCAGGAATTCGATCCCCAATTGATGGAGGACGCCAAGAA	Fw FLuc
oNH016	CGGGATCCCCCGGGCTGCAGGAATTCGATCCCCAATTGATGGGCGTCAAGGTG	Fw GLuc
oNH020	CGGGATCCCCCGGGCTGCAGGAATTCGATCCCCAATTGATGACCAGCAAGGTCTAC	Fw RLuc
oNH048	CGGGATCCCCCGGGCTGCAGGAATTCGATCCCCAATTGATGGTGCTCGGTCCTT	Fw SEAP
oNH057	CTTGTACAGCTCGTCCATG	Rev eGFP
oNH058	ATGGTGTCGGAGCTCATC	Fw mKate2
oNH116	GCCGGGCGGCCGGCGCGCCGGCCGCTAGATCTTTAGGCGTAGTCGGGCACGTCGTAAGGGTAGAGCGGACCCTGCTGCTCGTTCTTGAGCAC	Rev RLuc
oNH117	GCCGGGCGGCCGGCGCGCCGGCCGCTAGATCTTTAGGCGTAGTCGGGCACGTCGTAAGGGTAGAGCGGACCCTGGTCACCACCGGCAC	Rev GLuc
oNH119	GCCGGGCGGCCGGCGCGCCGGCCGCTAGATCTTTAGGCGTAGTCGGGCACGTCGTAAGGGTAGAGCGGACCCTGGACGGCGATCTTGCC	Rev FLuc
oNH122	CATATGGCGGTGACCG	Rev mKate2
oNH123	ATGTTTGAACGATCGCCGGGCGGCCGGCGCGCCGGCCGCTTTAGACCTTTCTCTTCTTTTTTGGAGGCGCTTTCTTGTACAGCTCGTCCATG	Rev eGFP
oNH124	ATCTGCCCTCGAAACTCGGTCACCGCCATATGATGACCAAGAAGTTTGGCACGCTCACCATCTAGGCCGGTTCGGGTGCCTCGTTAAAACAGCTCTGGGGTTG	Fw pIRES
oNH125	CCGGTGAACAGCTCCTCGCCCTTGCTCACCATACAATTCGCTTTATGATAACAATCTGTGATTG	Rev pIRES
oNH126	GCCGGGCGGCCGGCGCGCCGGCCGCTAGATCTTTAGGCGTAGTCGGGCACGTCGTAAGGGTAGAGCGGACCCTGCTGCTCGTTCTTGAGCAC	Rev RLuc
oNH127	CGGTGAACAGCTCCTCGCCCTTGCTCACCATGATTATCATCGTGTTTTTCAAAGGAAAAC	Rev eIRES
oNH128	ATCTGCCCTCGAAACTCGGTCACCGCCATATGATGACCAAGAAGTTTGGCACGCTCACCATCTAGGCCGGTTCGGGTGCCTCGAGCAGGTTTCCCCAATG	Fw fIRES
oNH129	CCGGTGAACAGCTCCTCGCCCTTGCTCACCATGGAAAGGAAAGGTGCCGAC	Rev fIRES
oNH130	CCACCCAGCTCATCTCGAACATGGACATCGACGTCATCCTCGGTGGTG	Fw SEAP
oNH131	CGCCGGGCGGCCGGCGCGCCGGCCGCTAGATCTCTATCCAGGGTGGGCG	Rev SEAP
oNH132	GCCGGGCGGCCGGCGCGCCGGCCGCTAGATCTTTAGGCGTAGTCGGGCACGTCGTAAGGGTAGAGCGGACCCTGCTATCCAGGGTGGGCG	Rev SEAP
oNH135	AGGCTGGATCGGTCCCGGTGTCTTCTATGGAGGTCAAAACAGCGTGGATGGCGTCTCCAGGCGATCTGACGGTTCACTAAACG	Rev PhCMVmin
oNH136	CTCCATAGAAGACACCGGGACCGATCCAGCCTGGCGCGCCATGGTGAGCAAGGGCG	Fw GFP
oNH137	TATTAATAGTAATCAATTACGGGGTCATTAGTTC	Fw GFP
oNH139	CACGCTGTTTTGACCTCCATAGAAGACACCGGGACCGATCCAGCCTCAATTGATGGTGTCGGAGCTCATC	Fw mKate2
oNH140	AATGGGCGGTAGGCGTGTACGGTGGGAGGTCTATATAAGCAGAGCTCGTTTAGTGAACCGTCAGATCGCCTGGAGACGCCATCCACGCTGTTTTGACCTCC	Fw PhCMV
oNH141	CCAAATGTTTGAACGATCGCCGGGCGGCCCAATTGCTAGATGGTGAGCGTGCCAAACTTCTTGGTCATCATATGGCGGTGACCG	Rev mKate2
oNH142	GGCCGCCCGG	
oNH143	TCACCATAGCAGGCCTAGATGGCCCCTGCAGGCTCATGTTTGACAGCTTATCATCG	Fw nosT
oNH144	CTCATGTTTGACAGCTTATCATCG	Rev nosT
oNH152	TTGAACATCAAATCAACTACCTTACTCTATCACAATTGATGGTGAGCAAGGGCG	Fw eGFP
oNH153	TTGAACATCAAATCAACTACCTTACTCTATCACAATTGATGGTGTCGGAGCTCATC	Fw mKate2
oNH157	AGTGTGGCACTCGAATCCCCCTGCTCGAGAAGAATCCGACAGCCAAACCTC	Fw Pmfa1min
oNH158	GCCCGGCGATCGTTC	Fw nosT
oNH169	ACTAGTCAATAATCAATGTCAACATGGCGGTCCAAATGGGCGGTAGGCG	Fw PhCMVmin
oNH174	TTGCCAAATGTTTGAACGATCGCCGGGCGGCCGGCGCGCCGGCCGCTTTAGACGGCGATCTTGC	Rev FLuc

oNH175	ACAATCACAGATTGTTATCATAAAGCGAATTGGCGATCGCATGGAGGACGCCAAGAA	Fw FLuc
oNH177	ACGTGGTTTTCCTTTGAAAAACACGATGATAAGCGATCGCATGGAGGACGCCAAGAA	Fw FLuc
oNH178	TCAAGAAGACAGGGCCAGGTTTCCGGGCCCTCGCGATCGCTTACTGCTCGTTCTTGAGC	Rev RLuc
oNH179	AATAGGTGACCGGAGGTCGGCACCTTTCCTTTGCGATCGCATGGAGGACGCCAAGAA	Fw FLuc
oNH180	TTGCACGTTTTGTGTCATTGGGGAAACCTGCTGCGATCGCTTACTGCTCGTTCTTGAGC	Rev RLuc
oNH181	CTGGGGTGGGTACAACCCCAGAGCTGTTTTAAGCGATCGCTTACTGCTCGTTCTTGAGC	Rev RLuc
oNH184	CTCCATAGAAGACACCGGGACCGATCCAGCCTCAATTGATGGAGGACGCCAAGAA	Fw FLuc
oNH196	CACGCTGTTTTGACCTCCATAGAAGACACCGGGACCGATCCAGCCTCAATTGATGACCAGCAAGGTCTAC	Fw RLuc
oNH197	TTGCCAAATGTTTGAACGATCGCCGGGCGGCCCAATTGTTACTGCTCGTTCTTGAGC	Rev RLuc
oNH198	CTCCATAGAAGACACCGGGACCGATCCAGCCTGGCGCGCCATGGAGGACGCCAAGAA	Fw FLuc
oNH205	ATGGTGAGCAAGGGCG	Fw mCherry,eGFP
oNH612	TTAAAACAGCTCTGGGGTTG	Fw pIRES
oNH613	CAATTCGCTTTATGATAACAATCTGTGATTG	Rev pIRES
oNH614	GAGGGCCCGGAAAC	Fw eIRES
oNH615	TTATCATCGTGTTTTTCAAAGGAAAAC	Rev eIRES
oNH616	AGCAGGTTTCCCCAATG	Fw fIRES
oNH617	AAAGGAAAGGTGCCGAC	Rev fIRES
oNH623	CGGGATCCCCCGG	Fw IRES-fusion
oNH624	TTGCCAAATGTTTGAACGATCG	Rev IRES-fusion
oNH625	CGGGATCCCCCGGGCTGCAGGAATTCGATCCCCAATTGGACCGCCATGTTGACATTGATTATTGACTAGTTATTAATAGTAATCAATTACGGGGTCATTAGTTC	Fw P_{CMV}
oNH626	CGGGATCCCCCGGGCTGCAGGAATTCGATCCCCAATTGCTTCTCGAGCAGGGGG	Fw P_{OMA}
oNH627	TCACTTCTCGCCCGTTCTTTTGAACATCAAATCAACTACCTTACTCTATCACAATTGATGACCAGCAAGGTCTAC	Fw RLuc
oNH628	AATCCGACAGCCAAACCTCATCCACTCTCACTTTCACACTCTAACTTATACGATCACTTCTCGCCCGTTC	Fw Pmfa1min
oNH645	TTGCCAAATGTTTGAACGATCGCCGGGCGGCCGGCGCGCCGGCCGCTTTACTGCTCGTTCTTGAGC	Rev RLuc
oNH646	CTCCATAGAAGACACCGGGACCGATCCAGCCTGGCGCGCCATGACCAGCAAGGTCTAC	Fw RLuc
oNH647	TTGCCAAATGTTTGAACGATCGCCGGGCGGCCCAATTGTTAGACGGCGATCTTGC	Rev FLuc
oNH657	CTCCATAGAAGACACCGGGACCGATCCAGCCTGGCGCGCCATGGTGTCGGAGCTCATC	Fw mKate2
oNH658	TGTTTGAACGATCGCCGGGCGGCCGGCGCGCCCTAGATGGTGAGCGTGCCAAACTTCTTGGTCATCATATGGCGGTGACCG	Rev mKate2
oNH659	CTCCATAGAAGACACCGGGACCGATCCAGCCTCAATTGATGGTGAGCAAGGGC	Fw eGFP
oNH660	TTGCCAAATGTTTGAACGATCGCCGGGCGGCCCAATTGGGCCGCTTTAGACCTTTCTCTTCTTTTTTGGAGGCGCTTTCTTGTACAGCTCGTCCATG	Rev eGFP
oNH695	AAGATCAAGGGTGCCGGTGGTGACTAATTAATTAAAACTTCTCGCCCGTTCTTTTGAACATCAAATCAACTACCTTACTCTATCAGGCGCGCCATGGAGGACGCCAAGAA	Fw FLuc
oNH696	TGATTTGATGTTCAAAAGAACGGGCGAGAAGTGATCGTATAAGTTAGAGTGTGAAAGTGAGAGTGGATGAGGTTTGGCTGTCGGATTCTCCCTTATATCCTTGACGGTAC	Rev P_{OMA}
oNH715	AGGCCAAGAAGGGTGGCAAGATCGCCGTCTAAGCGATCGCGGCCGCCCGG	Fw nosT
oNH716	CGAGCTCGGTACGGGGGATCCACTAGTTCTAGCTCATGTTTGACAGCTTATCATCG	Rev nosT
oNH717	GAGGCCAAAAAAGATACCATAATAGGCCTGAGTTAATTAAATGGAGGACGCCAAGAA	Fw FLuc
oNH718	CATAGTACATCAGGCTACTAACTGTC	Fw P_{CRG}
oNH719	CTCAGGCCTATTATGGTATCTTTTTTG	Rev P_{CRG}
oNH720	CACGCGTCTCACCATAGCAGGCCTAGATGGCCCCTGCAGGCATAGTA	Fw P_{CRG}
oNH721	TTGAACATCAAATCAACTACCTTACTCTATCAGGCGCGCCATGGTGAGCAAGGGCG	Fw eGFP
oNH722	TTGAACATCAAATCAACTACCTTACTCTATCAGGCGCGCCATGGTGTCGGAGCTCATC	Fw mKate2

Table S3. Strains used in this work

Strain	Description / Genetic background	Transformed Plasmid*	Origin
**AB33**	Induction of filamentous growth by changing the nitrogen source		Brachmann et al., 2001 (ref [14] in main text)
**UMa486**	AB33_IP::P_{Otef}-eGfp-CbxR		Baumann et al., 2014 (ref [27] in main text) (Fig.3B, S3)
**UMa1987**	AB33_pep4D: P_{Otef}-mKate2-Tnos-NatR	pUMa2986	this work (Fig.3B, S3)
**UMa3212**	AB33_rrm4D_P_{CRG}-rrm4-gfp-linker-P2A-FLuc-HA-nosT-cbxR		Müntjes et al., 2020 (ref [15] in main text) (Fig.S2B)
**sLHNH005**	AB33_upp3D::P_{O2tef}::RLuc-HA-nosT-NatR	pLHNH030	this work (Fig.1, 3C, S1, S2A, S3)
**sLHNH006**	AB33_upp3D::P_{O2tef}::GLuc-HA-nosT-NatR	pLHNH031	this work (Fig.1, S1)
**sLHNH007**	AB33_upp3D::P_{O2tef}::SEAP-HA-nosT-NatR	pLHNH032	this work (Fig.1)
**sLHNH008**	AB33_upp3D::P_{O2tef}::FLuc-HA-nosT-NatR	pLHNH033	this work (Fig.1,3C, 4, S1, S2, S3)
**sNH039**	AB33_upp3D::P_{CRG}::FLuc-nosT-P_{O2tef}::RLuc-nosT-NatR	pNH054	this work (Fig.2, S2C)
**sNH005**	AB33_upp3D::nosT-NES-mKate2::$P_{hCMVmin}$-CMVenhancer(5'->3')-$P_{hCMVmin}$::eGFP-NLS-nosT-NatR	pNH012	this work (Fig. 3B)
**sNH006**	AB33_upp3D::nosT-NES-mKate2::$P_{hCMVmin}$-CMVenhancer(5'<-3')- $P_{hCMVmin}$::eGFP-NLS-nosT-NatR	pNH013	this work (Fig. 3B)
**sNH007**	AB33_upp3D::nosT-NES-mKate2::$P_{mfa1min}$-(prf1)4(5'->3')-$P_{mfa1min}$::GFP-NLS-nosT- NatR	pNH014	this work (Fig. 3B)
**sNH008**	AB33_upp3D::nosT-NES-mKate2:: $P_{mfa1min}$-(prf1)4(5'<-3')-$P_{mfa1min}$::GFP-NLS-nosT-NatR	pNH015	this work (Fig. 3B)
**sNH011**	AB33_upp3D::nosT-RLuc:: $P_{hCMVmin}$-CMVenhancer(5'->3')-$P_{hCMVmin}$::FLuc-nosT-NatR	pNH030	this work (Fig. 3C)
**sNH012**	AB33_upp3D::nosT-RLuc:: $P_{hCMVmin}$-CMVenhancer(5'<-3')-$P_{hCMVmin}$::FLuc-nosT-NatR	pNH031	this work (Fig. 3C)
**sNH013**	AB33_upp3D::nosT-RLuc:: $P_{mfa1min}$-$(prf1)_4$(5'->3')-$P_{mfa1min}$::FLuc-nosT-NatR	pNH032	this work (Fig. 3C)
**SG200**	Solopathogenic strain		Kämper et al., 2006 (ref [7] in main text) (Fig.4)
**SG200-FLuc (UMa3062)**	SG200_upp3D::P_{O2tef}::FLuc-HA-nosT-NatR	pLHNH033	this work (Fig.4)
**SG200-pit1Δ**	SG200_ pit1D-HygR		Doehlemann et al., 2011 (ref [23] in main text) (Fig.4)
**SG200-pit1Δ-FLuc**	SG200_ pit1D-HygR_upp3D::P_{O2tef}::FLuc-HA-nosT-NatR	pLHNH033	this work (Fig.4)

**(UMa3132)**			
**sNH001**	AB33_upp3D::P_{O2tef}::RLuc-pIRES-FLuc-nosT-NatR	pNH026	this work (Fig.S3B)
**sNH003**	AB33_upp3D::P_{O2tef}::RLuc-eIRES-FLuc-nosT-NatR	pNH028	this work (Fig.S3B)
**sNH004**	AB33_upp3D::P_{O2tef}::RLuc-fIRES-FLuc-nosT-NatR	pNH029	this work (Fig.S3B)
**sLHNH009**	AB33_upp3D::P_{O2tef}::mKate2-NES-pIRES-eGFP-NLS-nosT-NatR	pNH009	this work (Fig.S3C,D)
**sLHNH010**	AB33_upp3D::P_{O2tef}::mKate2-NES-eIRES-eGFP-NLS-nosT-NatR	pNH010	this work (Fig.S3C,D)
**sLHNH011**	AB33_upp3D::P_{O2tef}::mKate2-NES-fIRES-eGFP-NLS-nosT-NatR	pNH011	this work (Fig.S3C,D)
